# Physical activity and sedentary behaviour of female adolescents in Indonesia: A multi-method study on duration, pattern and context

**DOI:** 10.1016/j.jesf.2022.02.002

**Published:** 2022-02-24

**Authors:** Fitria Dwi Andriyani, Stuart J.H. Biddle, Aprida Agung Priambadha, George Thomas, Katrien De Cocker

**Affiliations:** a Physically Active Lifestyles Research Group (USQ PALs), Centre for Health Research, University of Southern Queensland, Springfield Central, QLD, 4300, Australia; b Department of Sports Education, Faculty of Sports Science, Yogyakarta State University, Yogyakarta, 55281, Indonesia; c Department of Primary Teacher Education, Faculty of Teacher Training and Education, Ahmad Dahlan University, Yogyakarta, 55191, Indonesia; d Department of Movement and Sports Sciences, Faculty of Medicine and Health Sciences, Ghent University, Ghent, B9000, Belgium; e Curtin School of Allied Health, Faculty of Health Sciences, Curtin University, Perth, 6845, Australia

**Keywords:** LMIC, Non-screen-based sedentary behaviour, Screen time, Wearable camera, Youth

## Abstract

**Background/Objective:**

Exploring comprehensive information on the duration, pattern and context of physical activity and sedentary behaviour is important to develop effective policies and interventions. Especially in lower- and middle-income countries, our understanding of these health-behaviours is limited. Our study aimed to investigate physical activity and sedentary behaviour of female Indonesian adolescents by using a multi-method approach.

**Methods:**

Female adolescents (n = 5; 13–15 years old) from Yogyakarta, Indonesia wore accelerometers and automated wearable cameras for four days, and completed diaries, and interviews between February and March 2020.

**Results:**

Participants’ activity, especially on non-school days, was dominated by light-intensity physical activity. Four of the 5 participants did not meet the physical activity guidelines. Participants spent a great proportion of time on screen-based sedentary behaviour (school days: 83.2% of wear time; non-school days: 75.7% of wear time). During school days, most physical activity and sedentary behaviour was done at school. Screen time was mainly done on the school day evenings and weekend mornings. Participants mostly used smartphones in the bedroom and living room in a solitary environment. Interviews suggest that the high amount of screen time seemed to be influenced by a lack of awareness of current guidelines, the feeling of urgency to check information, and the lack of parental supervision. Non-screen-based sedentary behaviour comprised just over 10% of total camera images.

**Conclusion:**

The use of a multi-method approach facilitated a rich understanding of the duration, patterns, and contexts of physical activity and sedentary behaviour in participants. Future studies might consider using similar methods in a larger sample.

## 1 Introduction

More than 2.1 billion young people (<20 years old) worldwide are affected by non-communicable diseases (NCDs), including cardiovascular diseases, poor mental health, and diabetes.^1^ Among the key drivers of NCDs in youth are insufficient physical activity and high sedentary behaviour.^1^^,^^2^ Therefore, it is important to encourage youth to do more physical activity and limit excessive sedentary behaviour to maintain or enhance their health status and minimise the risk of NCDs. Specifically for this age group, studies show that physical activity has a positive association with better body mass index (BMI) profiles, physical fitness, bone health, cognitive function, academic achievement, and cardiometabolic biomarkers.^3^^,^^4^ Conversely, research has found that sedentary behaviour can be correlated with deleterious health outcomes, such as unfavourable BMI, lower fitness, psychosocial health, and sleep quality as well as higher cardiometabolic risk.^4^, ^5^, ^6^ Nevertheless, increasing physical activity level and reducing sedentary behaviour in youth may be challenging, especially when the knowledge concerning young people's physical activity and sedentary behaviour is still somewhat limited, especially in lower-middle-income countries (LMICs).^7^, ^8^, ^9^

Physical activity and sedentary behaviour research in youth has been conducted almost exclusively in high-income countries. Investigations are scarce in LMICs,^10^^,^^11^ including Indonesia.^7^ As a country with around 46 million adolescents (age 10–19),^12^ and with a mortality rate due to NCDs reaching 71%,^13^ it is important to conduct more research on these topics in Indonesia. A previous national study showed that 6 out of 10 Indonesian adolescents had insufficient physical activity.^14^

Moreover, previous studies have largely focused on one aspect of behaviour, such as physical activity (e.g., see Crooks et al.^15^), and often neglect non-screen-based sedentary behaviours in investigations. Researching physical activity, as well as screen- and non-screen-based sedentary behaviour, may provide a better understanding of young people's behaviour. Exploring such broader information is important as it may assist the formulation of more effective policies and interventions to increase physical activity and to limit excessive sedentary behaviour in youth.

Measurement is an important aspect of investigations concerning the nature and level of physical activity and sedentary behaviour in youth. There has been a range of methods available to assess both behaviours, including self-report questionnaires and diaries, and technological devices, such as accelerometers and automated wearable cameras. Each instrument has some strengths as well as limitations (see Barnett et al.^16^). Consequently, it should be advantageous to use multiple assessment methods in the same study and triangulate findings.

The American Heart Association points out that there is a need to gather more accurate information on the nature of behaviour by using both quantitative and qualitative methods.^16^ As such, we sought to investigate physical activity and sedentary behaviour in youth by using a comprehensive multi-method approach. We used different methods, involving device-based and self-reported measurement as well as quantitative and qualitative methods to investigate both physical activity and sedentary behaviours. Specifically, we used accelerometers to measure duration, intensity, and patterns of activity; automated wearable cameras to capture types and contexts of behaviour; diaries to record missing data when participants failed to wear the devices; and interviews to explore reasons and contexts of youth's activity. We claim this combination of methods to be highly novel.

Our study focused on adolescents as this age group has a high prevalence (81%) of insufficient physical activity^17^ and is the most sedentary across pediatric populations.^16^ Specifically, our study focused only on female adolescents for several reasons. First, this group is often identified as less active than their male counterparts.^11^^,^^17^ Second, girls are known to have different patterns of screen use and sedentary time from boys.^18^^,^^19^ Further, girls were found to have some different correlates of physical inactivity and sedentary behaviour from boys.^20^ Thus, our study aimed to provide an in-depth understanding of physical activity and sedentary behaviours in a small group of female Indonesian adolescents by using a comprehensive multi-method approach to capture behavioural quantity and patterns, context, and underlying biopsychosocial perceptions and correlates.

## 2 Methods

### 2.1 Study design

This study employed a mixed-method approach, particularly the convergence model.^21^ In this model, the researchers collected both quantitative and qualitative data followed by examining both data to develop a better understanding of the findings.^21^ We collected data by using accelerometers, automated wearable cameras, diaries, and interviews. Data were collected in Yogyakarta, Indonesia, from February to March 2020, and were analysed from April 2020 to May 2021. Data were collected on days agreed by participants and school principals and those days were on non-Physical Education (PE) school days.

### Ethical approval

Ethical approval for our study was obtained from the Human Research Ethics Committee of the University of Southern Queensland (approval number: H19REA221).

### 2.2 Participants

Due to the intensive, burdensome, and somewhat intrusive nature of data collection for participants, we restricted recruitment to 5 female adolescents (age 13–15 years old) from one public (government-funded) school in an urban area of Yogyakarta Province, Indonesia. We explained our study to a PE teacher who then passed on this information to her students when teaching PE classes (two classes). The teacher delivered our invitation to female students to attend an information session to get further details of our study. Among six female students who expressed their interest, five attended the information session, at the end of which the lead author (FDA) answered questions and provided research packs to the students. The lead author also made a phone call to the parents of the prospective participants to explain the study and answered their questions. The five female students and their parents agreed to participate in the study. Written parental and adolescent consent was obtained from all participants. Participants were required to have access to a smartphone during the study so that we were able to remind and encourage them to follow our study protocol.

### 2.3 Measures

#### 2.3.1 Sociodemographic questionnaire

Before the main data collection commenced, we asked participants and their parents to complete a brief questionnaire concerning demographic characteristics, including education level, occupation, number of screen-based devices at home, and the most used social media application (app).

#### 2.3.2 Anthropometry

Height was measured to the nearest 0.1 cm using a wall-mounted stadiometer. Body weight was determined to the nearest 0.1 kg using a digital scale. Participants took off their shoes when being measured. Weight status was determined using sex and age-specific body mass index (BMI) standards of the World Health Organization (WHO).^22^

#### 2.3.3 Accelerometers

To assess physical activity and sedentary time, we used the ActiGraph wGT3X-BT accelerometer (ActiGraph, LLC, Florida; hereafter ‘ActiGraph’). This device has dimensions of 3.3 × 4.6 × 1.5 cm with a weight of 19 g. It is a 3-axis accelerometer with a dynamic range of ± 8 G. Acceleration data are sampled by a 12-bit analog-to-digital converter with sample rates ranging from 30 Hz to 100 Hz. Using a sample rate of 30 Hz, it has a battery life of around 20 days. Participants were asked to wear the accelerometer during waking hours for four days (three school days, one non-school day). Donaldson et al.^23^ reported that four days of accelerometer measurement would be comparable to one week for estimating sedentary time.

We followed the protocol for wearing and processing ActiGraph data for children and adolescents by Chandler et al.^24^ They found a favourable accuracy of activity intensity classification, with Receiver Operator Characteristic (ROC) analyses resulted in an area under the curve (AUC) ranged 0.82–0.89, 0.80–0.83, 0.62–0.67 and 0.86–0.89 for light, moderate, vigorous, and moderate-to-vigorous physical activity intensities, respectively.^24^ The accelerometers were set to collect data by using a 5-s epoch length. Participants wore the ActiGraph on their non-dominant wrist. Previous studies found a favourable comparison in average activity between waist-worn and wrist-worn accelerometers^25^ and that children/adolescent participants reported higher compliance for wrist-worn compared to hip-worn accelerometers.^25^^,^^26^ Accelerometer placement on the non-dominant wrist was also suggested to minimise “noise” during certain sedentary behaviours.^27^ Participants were instructed to remove the device when doing water activities (e.g., taking a shower, swimming).

Our study used a sampling frequency of 30 Hz. Data showed that the majority of previous accelerometer studies in children and adolescents used a sampling frequency of 30 Hz and that the initial filtering process was developed for 30 Hz by the manufacturer.^28^ The time of the ActiGraphs was synchronized with the time of the wearable cameras (see later). The filter of the accelerometer was set to normal rather than LFE (low frequency) filter. Migueles et al.^28^ explained that enabling the LFE filter compared with the normal filter will result in showing decreased sedentary time, greater time in physical activity at all intensities, and an increase in the number of steps per day.

#### 2.3.4 Accelerometer data processing

Our study used Vector Magnitude, which is the square root of the sum of squared activity counts from the three axes, for data processing to be consistent with previous studies.^28^ The ActiLife Software (v6.13.4) was used to process data. To clean the data, non-wear time was defined as 20 min of consecutive zeros of the ActiGraph count per minute. This criterion has been used in the majority of research using accelerometers.^28^ Cut points for classifying the intensity of activity followed Chandler et al.^24^ (See Table 1). Data cleaning and processing were conducted between April and June 2020. Details of the manual for accelerometer data processing is available on request from the first author.

**Table 1:** Sedentary time and physical activity intensity classification for children and adolescents based on Chandler et al.^24^

Axis	Intensity Category	Counts per 60 s
Vector Magnitude	Sedentary	<3660
	Light physical activity	3660–9815
	Moderate physical activity	9816–23628
	Vigorous physical activity	>23,628

#### 2.3.5 Automated wearable cameras

Our study utilised Brinno TLC120 automated wearable cameras (Brinno Inc, Taiwan). The camera has dimensions of 60 × 60 × 33.5 mm with a weight of 101 g. The camera automatically takes a photo from the participant's point of view every ∼10 s. Using this setting, the camera has a battery life of 6 days. The device took images and converted them automatically to a time-lapse video (.avi) and stored it on an SD card. It created a new video every time the on/off button was pressed. The videos then were manually converted into single images (.jpg) using the open-source software FFmpeg (version 4.3).

Our study followed the ethical framework for human research using wearable cameras by Kelly et al.^29^ Participants were instructed to wear the camera on an adjustable chest mounted harness. Simultaneously with the data collection of the accelerometer, participants were asked to wear the camera during their free time, and specifically on three school day evenings, after school hours until bedtime, and during waking hours on one non-school day. Participants were instructed to remove the device when they were in situations that needed privacy (e.g., restroom, sports changing room). Third parties were able to ask participants to switch off the camera and ask for deletion of their images by contacting the researcher. A reference card was provided for each participant, which contained a statement for anyone with questions about the device and the contact information of the researcher.

Participants and one of their parents had an opportunity to review and delete any collected images prior to the lead author viewing and analysing the images. For privacy reasons, the researcher was required not to provide a copy of images under any circumstances. To protect participants’ privacy, the images remaining after any deletions were securely stored in a password-protected device and on a password-protected storage server. Images could only be accessed by the lead author (F.D.A.), who acted as the main image coder, and one other researcher (A.A.P.) who acted as a second image coder. The second image coder was only able to access a subset of images (10%), as set by the lead author, to check for coding agreement.

#### 2.3.6 Camera data processing

Image coding was completed between September 2020 and April 2021. With the help of other collaborating researchers,^30^^,^^31^ we developed a guideline to code the images (see Appendix A). Participants’ images were manually coded by the lead author using Excel spreadsheets. The guideline was refined during image coding.

Images were coded into 17 categories. The main behaviour was categorised into screen-based sedentary behaviour, non-screen-based sedentary behaviour, physical activity, or screen-based physical activity. Posture was coded into five different categories (sitting/lying/reclining, standing still, standing with movement, walking/running, and bicycling). The use of technological devices (e.g., smartphones) was classified in terms of the extent of the attention that they appeared to require. Coding was therefore as primary, secondary, background, and unclassifiable.

The device was categorised into portable (e.g., smartphone, laptop), non-portable (e.g., television, desktop computer), and wearable (e.g., smartwatch). Content types were classified into passive and interactive screen media, and social media. Passive screen media involves sedentary activities, with the user mainly receiving screen-based content passively,^32^ such as watching programs on TV or videos on YouTube. Meanwhile, interactive screen media requires cognitive or physical engagement during screen use, such as doing schoolwork on a laptop and playing video games.^32^

**Fig. 1:**
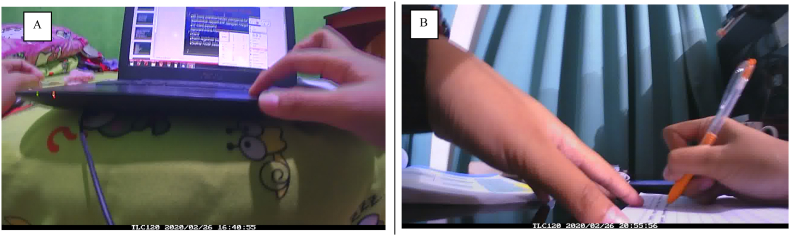
Sample of Images and Coding A. Date: February 26, 2020; Time: 16:40:55; Main behaviour: screen-based sedentary behaviour; Posture: Sitting; Device attention: primary; Device: Portable:Laptop computer; Content type: interactive screen media:creation:writing app (e.g., PowerPoint); Purpose: educational; Physical setting: home:bedroom; Social context: alone; Social environment: alone; Social interaction: none; Other behaviour: none. B. Date: February 26, 2020; Time: 20:55:56; Main behaviour: non-screen-based sedentary behaviour; Posture: Sitting; Device attention: none; Device: none; Content type: none; Activity: writing:doing schoolwork; Purpose: educational; Physical setting: home:bedroom; Social context: alone; Social environment: alone; Social interaction: none; Other behaviour: none.

Blurry or blocked images, and images with poor lighting, were coded as uncodeable or coded based on the preceding and subsequent images. Date, time, image number, activity, purpose, physical setting, social context, social environment, social interaction, other behaviours, and other devices in use (if any) were also coded. Examples of images and coding are presented in Fig. 1.

Percent agreements between the main and the second coder were checked across ∼10% of the images (n = 2095 images) and across 8 coding categories (main behaviour, posture, device, content type, activity, purpose, physical setting, and social interaction). On average, we found 99% agreement. We calculated the percent agreement by using the following formula:^33^

$$P_{A}\;=\;\frac{N_{A}}{N_{A}+N_{D}}\times 100$$

Note:

P_A_ = Percent agreement.

N_A_ = Total number of agreements.

N_D_ = Total number of disagreements.

#### 2.3.7 Diary

Each participant was provided with a pre-formatted diary (small logbook), during data collection. The diary was used to record activities only whenever participants removed the wearable camera and/or the accelerometer, such as when doing water activities. Specifically, it recorded date, what device than has been taken off (e.g., accelerometer and camera), time when taking off the device (e.g., 15.00–15.30), and activity when taking off the device. It was also used to record bedtime and wake up time.

#### 2.3.8 Interviews

A one–on–one semi-structured interview (mean duration: 28 min) was conducted once for participants. The interviews were conducted at their school in March 2020 by the lead author (F.D.A). These interviews were used to investigate the contexts and reasons for participants’ physical activity and sedentary behaviour (screen- and non-screen-based). Interviews were audio-recorded using Olympus DS-3500 (Olympus Imaging Corp, China) and Olympus ME33 (Olympus Imaging Corp, Taiwan). The interview guideline can be seen in Appendix B.

#### 2.3.9 Data Analysis

We used Microsoft Excel for Microsoft 365 MSO (16.0.14228.20200) for descriptive analyses. Diary data were incorporated into the accelerometer data to capture any missing activities when participants removed the devices during waking hours. The activity captured by the diary were converted into the proper intensity by following the 2011 Compendium of Physical Activities.^34^ Diary and accelerometer data were also cross-checked to confirm accelerometer non-wear time during waking hours. For accelerometer combined with diary data, descriptive data were provided to describe the length of sedentary behaviour, light through to vigorous intensity of physical activity, and device wear time on both school day and non-school day. We reported the accelerometer data by using data on vector magnitude.

To analyse temporal patterning, we divided the accelerometer combined with diary data on school days into different time segments: before school (before 07.00 h), school (07.00 h–15.00 h), late afternoon (15.00 h–18.00 h), and evening (18.00 h to sleep). On the non-school days, we divided the data as follows: morning (before 12.00 h), early afternoon (12.00 h–15.00 h), late afternoon (15.00 h–18.00 h), and evening (18.00 h to sleep).

For camera data, descriptive data were provided to describe the total number of images, camera wear time, captured time, and the number of images and duration for physical activity, screen-based physical activity, screen-based sedentary behaviour, and non-screen-based sedentary behaviour on both school days and non-school days. We defined camera wear time as the total number of minutes the camera was turned on. Captured time (in minutes) was defined as the number of images divided by 6 (assuming each image representing 10 s). Descriptive data described the types of devices being used, the content, and the social and environmental context of the behaviours. To analyse the temporal patterning of screen-based behaviours between the different evening segments, we divided screen time data into two segments during the school day: late afternoon (15.00 h–18.00 h) and evening (18.00 h to sleep). For non-school day, we divided screen time data into different segments as follows: morning (before 12.00 h), early afternoon (12.00 h–15.00 h), late afternoon (15.00 h–18.00 h), and evening (18.00 h to sleep).

Interview recordings were transcribed verbatim and anonymised. Transcripts were imported into NVivo software (Version 12 Pro, QSR International, Victoria, Australia) to facilitate analysis. The transcripts were analysed by using the reflexive thematic analysis approach as follows: 1) Familiarisation with the data, 2) Coding, 3) Generating initial themes, 4) Reviewing themes, 5) Defining and naming themes, and 6) Writing up.^35^^,^^36^ Firstly, the lead author (F.D.A.), who interviewed the participants, became immersed in the dataset by listening to the interview recordings and reading the transcripts. Afterwards, she coded the transcripts, followed by generating initial themes by using the inductive approach to avoid the theme's production being influenced by the theoretical interest of the researcher^37^ that may affect the analysis. Finally, all authors critically reviewed themes, including defining and naming themes before writing up the results. Data were analysed between May and August 2021. We triangulated the results of interviews, accelerometers, automated wearable cameras, and diary data.

## 3 Results

Results are presented separated and combined by the measurement method. Qualitative data are reported alongside that from devices to allow for better integration and triangulation of findings.

### 3.1 Sample characteristics

The characteristics of the participants are presented in Table 2. Five female adolescents participated in our study, with an average age of 13.9 ± 0.4 years old. The majority of the participants had normal body mass index (BMI) and the average number of people in the participant's household was five. All participants reported having access to television (TV), laptop, and smartphone. WhatsApp, Instagram, and YouTube were the most popular social media platforms used by participants.

**Table 2:** Characteristics of the sample (*n* = 5).

Variables	Total
Gender (% female)	100%
Age (Mean *SD*; years)	13.9 (0.4)
Height (Mean *SD*; cm)	156.5 (4.9)
Weight (Mean *SD*; kg)	54.1 (14.1)
BMI (Mean *SD*; kg/m^2^)	21.9 (4.3)
Parent Age (Mean *SD*; years)	43.5 (8)
Number of People in Household (Mean *SD*)	5 (3)
Parents' Highest Level of Education (*n*, %)	
*Doctoral Degree*	1 (20%)
*University or Tertiary Qualification*	2 (40%)
*Year 12 or Equivalent*	2 (40%)
Parent Occupation (*n*, %)	
*Entrepreneur*	1 (20%)
*Housewife*	2 (40%)
*Teacher*	1 (20%)
*Lecturer*	1 (20%)
Number of screen-based devices at home (Mean *SD*)	
*Television*	1 (0.4)
*Desktop computer*	0.2 (0.4)
*Laptop*	1 (0.5)
*Smartphone*	4 (1.5)
*Video games that connected to TV (*e.g.*, Playstation)*	0.2 (0.4)
*Portable games player (*e.g.*, Nintendo DS)*	0.4 (0.5)
The most used social media application (*n*, %)	
*WhatsApp*	5 (100%)
*Instagram*	5 (100%)
*YouTube*	4 (80%)
*TikTok*	3 (60%)
The most used social media application by parents (*n*, %)	
*Facebook*	5 (100%)
*WhatsApp*	5 (100%)
*Instagram*	3 (60%)

Notes: BMI = Body Mass Index; SD = Standard Deviation.

Due to unexpected circumstances, three participants wore the accelerometer and the camera for 2 school days and 2 non-school days; one participant wore the devices on 3 schooldays and 1 non-school day; and one participant wore the devices on 1 school day and 2 non-school days. Three participants initially provided incomplete data and two of them agreed to wear the devices again to complete the data.

The average wear time of the accelerometer during the school day was significantly higher than that of non-school day, 743 ± 100.1 min/day vs 347 ± 175.8 min/day (∼12.4 ± 1.7 h/day vs 5.8 ± 2.9 h/day) respectively. Participants wore the camera simultaneously on the days they wore the accelerometer, with a total wear time of 55.5 h. A total of 19,942 images, derived from 10 school days and 9 non-school days, were coded and included in the analysis. On average, the camera captured 683 images (∼114 min of captured time) on each school day and 1457 images (∼243 min of captured time) on a non-school day.

### 3.2 Duration of physical activity and sedentary behaviour

Data from the accelerometer combined with the diary (see Data Analysis) showed that, on average, participants spent a great proportion of their time in sedentary behaviours: 518 min (69.7% of wear time) on a school day and 282 min (81.3% of wear time) on a non-school day. Only one participant met the WHO's physical activity guideline for adolescents, which is accumulating at least an average of 60 min per day of moderate-to-vigorous physical activity across the week^38^ (in this study, the average was calculated across the 4-day data collection). Participants' physical activity was dominated by light intensity: 180 min (24.2% of wear time) on a school day and 61 min (17.6% of wear time) on a non-school day. Details of the accelerometer combined with diary data can be seen in Table 3.

**Table 3:** Summary accelerometer combined with diary data.

Variable	Minimum	Maximum	Median	Mean
School day (in minutes)				
**Wear time**	576	820	777	743
**SB**	443	619	501	518
**LPA**	130	229	169	180
**MPA**	2	19	5	8
**VPA**	0	185	0	37
Non-school day (in minutes)				
**Wear time**	145	609	353	347
**LPA**	14	116	65	61
**MPA**	0	9	4	4
**VPA**	0	0	0	0

Notes: SB = Sedentary Behaviour; LPA = Light Physical Activity; MPA = Moderate Physical Activity; VPA = Vigorous Physical Activity.

From the wearable camera, we found similar results as the most captured images contained screen-based sedentary behaviour. On average, participants engaged in screen-based sedentary behaviour for 94.8 min (83.2% of wear time) after school on a school day, and 184 min (75.7% of wear time) on a non-school day. The majority of participants did little physical activity while wearing the camera, averaging 2 min (1.9% of wear time) on a school day after school and 13 min (6.6% of wear time) on a non-school day. Details of images and camera wear time are presented in Table 4.

**Table 4:** Frequency of images and camera wear time per day.^a^.

Variable	School day (*n* of images = 6826)				Non-school day (*n* of images = 13,116)			
	Minimum	Maximum	Median	Mean	Minimum	Maximum	Median	Mean
**Number of images**	78	1395	624.5	683	455	2951	1373	1457
**Time of first image, h:min:s**	16:00:00	23:00:00	17:54:00	18:19:00	7:37:00	13:29:00	9:28:00	10:02:40
**Time of last image, h:min:s**	18:11:00	23:36:00	20:17:00	20:30:12	13:00:00	21:46:00	19:54:00	18:20:13
**Wear time, min**^b^	13	233	104	114	76	495	229	243
**Captured time, min**^c^	13	233	104	114	76	492	229	243
**PA images**	0	32	11	12	0	287	63	80
**PA, min**^c^	0.0	5.3	1.9	2.0	0	48	10.5	13
**Screen-based PA images**	0	5	0	0.7	0	110	4	19
**Screen-based PA time, min**^c^	0	0.8	0	0.11	0	18	1	3
**Screen-based SB images**	0	1308	593.5	568.8	214	2283	846	1104
**Screen time, min**^c^	0	218	98.9	94.8	36	381	141	184
**Non screen-based SB images**	6	255	50.5	78.2	14	348	127	153
**Non screen-based SB time, min**^c^	1	42.5	8.45	13.04	2	58	21	25
**Uncodeable images**	0	71	13	23	0	351	23	101

Notes.

h, hour; min, minute; s, second; PA, physical activity; SB, sedentary behaviour.

a Included 19 days (10 schoolday evenings and 9 non-school days) from 5 participants.

b Minutes the camera was turned on.

c One image represents 10 s (number of images/6).

Device-based measurements showed that most participants did a small amount of moderate-to-vigorous physical activity. Based on the interviews, this finding seemed partly to be associated with the fact that none of the participants joined extracurricular sports activities at school and most of them did not join any sports clubs in the community. Data from the diary and interviews showed that only one participant did any vigorous physical activity, done while attending a sports club.

Device-based data also showed that participants did more sedentary behaviour on weekends than weekdays (see Table 3, Table 4). This was also confirmed in interviews where all participants reported accessing screen-based devices more during the weekend than weekdays as they have more opportunities to do so.

“(In weekend days) I take a rest at home, usually watched TV, then also use a smartphone in between” **(PF2).**

“(I used my smartphone) until 2 a.m. on weekend days” **(PF4).**

### 3.3 Patterns of physical activity and sedentary behaviour

**Fig. 2:**
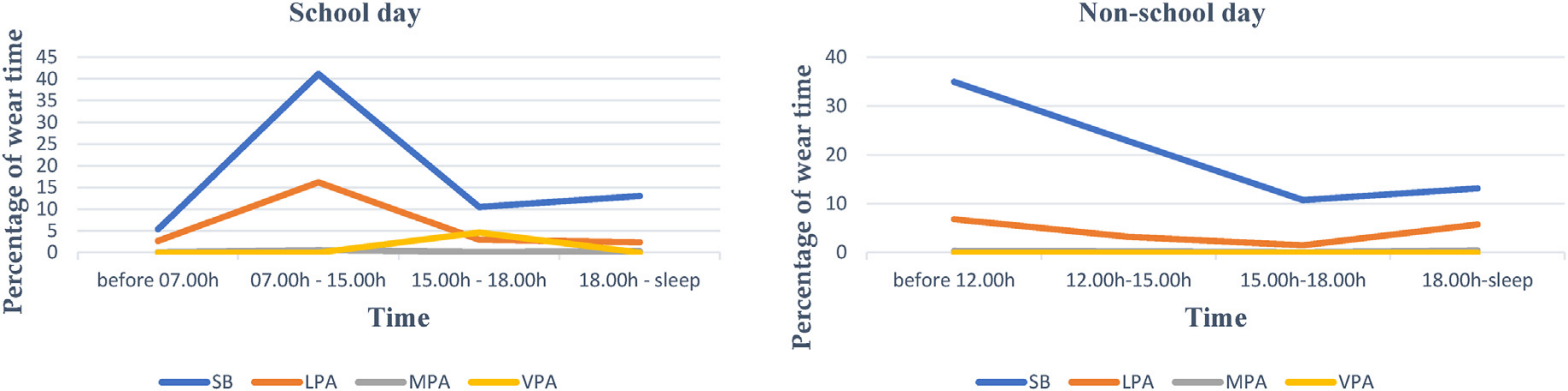
Patterns of activity during school and non-school days using accelerometer data Note: SB = Sedentary behaviour; LPA = Light physical activity; MPA = Moderate Physical Activity, VPA = Vigorous Physical Activity.

Fig. 2 shows the pattern of participants’ activity for the school and non-school days based on movement intensity by using accelerometers combined with diary data. During school days, high proportions of sedentary behaviour (40% of wear time) but less for physical activity (16%) were done at school. After school, participants did slightly more sedentary behaviour in the evenings than in the late afternoon (13.1% vs 10.5% of wear time). The little vigorous physical activity that was done was performed directly after school time until late afternoon, but this was only done by one participant. On non-school days, participants engaged in sedentary behaviour in the morning, with about 35% of wear time. This rate then gradually decreased during the afternoon, reaching ∼11% of wear time, and had a small increase in the evening (just over 13% of wear time). The pattern of moderate and vigorous physical activity was about the same across the day, and close to 0% of wear time.

**Fig. 3:**
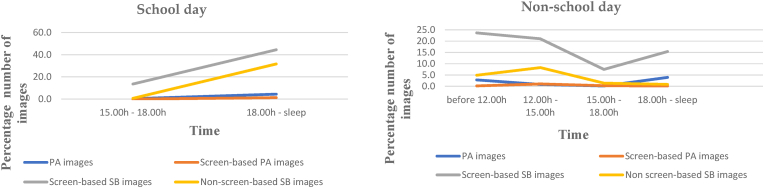
Patterns of behaviour on school and non-school days using camera data Note: PA = Physical activity; SB = Sedentary behaviour.

Fig. 3 illustrates the patterns of participants' activity based on the number of camera images for both school and non-school days. On average, participants’ images were dominated by screen-based sedentary behaviour images. On school days, in line with the accelerometer and diary data, participants did more screen-based sedentary behaviour in the evening than in the late afternoon (44.4% vs 13.6% number of images), as expected. Non-screen-based sedentary behaviour, such as writing, doing homework, and drawing, was mainly done in the evening (31.6% number of images). On non-school days, consistent with the accelerometer and diary data, participants did screen-based sedentary behaviour more in the morning (23.7% number of images). Related to the accelerometer data trends, images containing physical activity were minimal across wear time (less than 5% number of images), both on school and non-school days. More details of Fig. 2, Fig. 3 can be seen in Appendix C, Table C1 and C2.

Interviews revealed that participants spent a large proportion of their daytime at school during school days, from just before 7 a.m. to around 3 p.m. and even more when they attended extracurricular activities. This may explain findings related to patterns from accelerometer data, which showed that participants did most of their physical activity and sedentary behaviour at school during weekdays. However, it is important to note that those results might be caused by the fact that participants wore the accelerometer much longer during school time (∼58% of wear time) than after school (34% of wear time). Camera data showed the patterns of both screen- and non-screen-based sedentary behaviour increased steadily from after school to evening, reaching ∼45% and ∼32% number of images respectively.

“(I go back home) depends on the day. On Monday (I go back home) at 2.30 pm, Tuesday at 6 pm, Wednesday at 2.30 pm, Thursday at 6 pm, Friday at 2 pm (**PF2**).

“(I go back home) at 5.30 pm. School finished at 2.30 pm, but I go back home at 5.30 pm (I: What did you do?) Mm … usually because of my own will and I had a meeting (with friends) for school organization. For last week, (I go back at 5.30 pm) 4 times, for school organization and extracurricular activities” **(PF5).**

Note: I: Interviewer; P: Participants.

On non-school days, device-based data showed that participants did most of their screen time in the morning. The fact that there were many cartoon programs aired on TV on weekend mornings may have caused this. Interviews revealed that participants watched cartoon programs either on TV or YouTube and showed they liked them.

### 3.4 Types, contents, and purposes of physical activity and sedentary behaviour

Based on camera data, we found that participants allocated the least proportion of time for physical activity (5.1% of total images). Of this, more than a quarter of images consisted of domestic work (27.9%). This finding is consistent with interview data where participants reported some forms of light-intensity physical activity by doing domestic work, such as sweeping the floor, folding and ironing clothes, especially on weekends. In interviews, all participants cited that they did physical activity through PE lessons. This was not captured in accelerometers as they wore the devices on non-PE school days and not captured in cameras as they wore them after school hours. One participant joined a sports club. This participant removed the devices during her training, so we obtained her training data through the diary and interview. Some participants also mentioned doing some forms of physical activity such as dancing with music on YouTube, doing exercise (e.g. push-ups), playing badminton, and bicycling.

“Yes, sometimes (I) swept the floor while listening to music using a headset, sometimes it made me not sweeping the floor but just dancing following the song” **(PF3).**

Non-screen-based sedentary behaviour comprised just over 10% of total images. The highest proportion of non-screen-based sedentary behaviour was for educational and leisure purposes (44.3% and 18%, respectively). These findings are also in line with interview results. Interviews revealed that only one participant was keen on doing non-screen-based sedentary behaviour for leisure activity, particularly for drawing animation figures.

Participants utilised more than three-quarters of the device wear time (78.4% of total images) for screen-based sedentary behaviour, particularly using a smartphone, laptop, and TV. Of these, participants mainly used smartphones (around 81% of screen time) and this is also confirmed in interviews. Among screen-based images, 60.2% contained passive screen time. We cannot classify around half of the smartphone content as the camera did not capture the content properly (e.g., images showed only a small part of the smartphone being used). From the rest of the identifiable data, we found that leisure dominated the purpose of smartphone use (30.1%). The purpose of screen use by using the laptop was mainly for educational purposes (77.5%), while TV watching was, as expected, mostly for leisure (98.5%). Details of types and contents of behaviours can be seen in Table 5, while details of the purposes can be seen in Appendix D, Table D1.

**Table 5:** Type and content of behaviours based on camera data.

Variable	*n* of images	%
**Movement behaviours**	1016	5.1
**Non-screen-based sedentary behaviour**	2155	10.8
**Screen-based sedentary behaviour**	15,628	78.4
**Uncodeable**	1143	5.7
SUM	**19,942**	**100.0**
Types of Movement behaviours		
**Physical Activity: walking**	181	17.8
**Self-care (e.g., taking a shower, brushing teeth)**	7	0.7
**Chore (e.g., cleaning house, cooking, washing dishes/clothes/vehicles)**	283	27.9
**Unclassifiable**	257	25.3
**Other**	107	10.5
**Standing and Eating Snack**	2	0.2
**Standing and Eating Meal**	4	0.4
**Screen-based Physical Activity**	175	17.2
SUM	**1016**	**100.0**
Types of Non-screen-based sedentary behaviour		
**Writing: Doing schoolwork**	766	35.5
**Writing: Other**	89	4.1
**Reading: Educational book**	189	8.8
**Reading: Novel**	58	2.7
**Reading: Other**	37	1.7
**Hobby: Art: Painting/Drawing/Colouring**	329	15.3
**Sitting/lying/reclining**	360	16.7
**Sitting and Eating Other**	15	0.7
**Sitting and Eating Meal**	97	4.5
**Sitting and Eating Snack**	19	0.9
**Drinking: Beverage**	19	0.9
**Socializing**	39	1.8
**Self-care (e.g., taking a shower, brushing teeth, and putting on makeup)**	2	0.1
**Other**	79	3.7
**Unclassifiable**	57	2.6
SUM	**2155**	**100.0**
Types of Screen-based sedentary behaviour (Content)		
Portable: Mobile Device (smartphone)		
**Unclassifiable**	6237	49.3
**Passive Screen Media: Programme: Animation/Cartoon**	80	0.6
**Passive Screen Media: Programme: Live Action Animation**	1213	9.6
**Passive Screen Media: Programme: Live Action**	907	7.2
**Passive Screen Media: Unclassifiable (e.g., watching/looking at something)**	382	3.0
**Passive Screen Media: Programme: Unclassifiable**	447	3.5
**Passive Screen Media: General (e.g., home page, lock screen notifications)**	5	0.0
**Interactive Screen Media: Creation: Camera App (e.g., photo/video)**	5	0.0
**Interactive Screen Media: Unclassifiable (e.g., scrolling, browsing)**	716	5.7
**Interactive Screen Media: Internet: Browse (e.g., scrolling, online shopping)**	104	0.8
**Interactive Screen Media: Other**	452	3.6
**Interactive Screen Media: Communication: Video Chat**	47	0.4
**Social Media: WhatsApp**	1918	15.2
**Social Media: Instagram**	137	1.1
SUM	**12,650**	**100.0**
Portable: Laptop Computer		
**Unclassifiable**	41	10.9
**Interactive Screen Media: Unclassifiable (e.g., scrolling, browsing)**	37	9.8
**Interactive Screen Media: Creation: Writing Apps (e.g., word, PowerPoint)**	292	77.5
**Passive Screen Media: General (e.g., home page, lock screen notifications)**	7	1.8
SUM	**377**	**100.0**
Non-portable: Television		
**Unclassifiable**	38	1.5
**Passive Screen Media: Programme: Unclassifiable**	519	20.0
**Passive Screen Media: Programme: Live Action**	1431	55.0
**Passive Screen Media: Programme: Animation/Cartoon**	613	23.5
SUM	**2601**	**100.0**

### 3.5 Physical setting

Camera data showed that most of the physical activity (39.7%) and non-screen-based sedentary behaviour (66.2%) were undertaken in the bedroom. Smartphone screen time was mainly done in the bedroom (50.5%) and living room (44.9%). While laptop use was predominantly done in the bedroom, TV watching was mostly in the living room (60.2%). Details of the physical setting can be seen in Appendix D, Table D2. Some participants mentioned that the main reason to use the smartphone in the house was because of the quiet environment.

“(I used phone) the longest at home because it is quieter. It is noisy right here (at school)” **(PF4)**.

### 3.6 Social context, environment, and interaction

Table D3 in Appendix D shows the social context, environment, and interaction of participants' activity based on camera data. The social context and environment for participants’ physical activity were mainly solitary, without social interaction (nearly 90%). Similar findings were found for non-screen based sedentary behaviour (just over 80%) and overall screen use (smartphone: more than 70%, laptop: 100%, TV: more than 60%). More than a quarter of images containing smartphone use and more than one-third of images containing TV watching showed social environments but with no interaction. From interviews, participants seemed to enjoy using their screen-based devices alone because they wanted freedom in accessing content.

“No, (I used screen-based devices) individually. If there is a family member wanting to watch TV, then he/she watches TV; others then use a phone. So, (we will) not fight **(PF3).**

### 3.7 Co-existing behaviour

From camera data, we found that co-existing behaviour did not really exist (nearly 100%) across all participants’ behaviour (physical activity, screen- and non-screen based sedentary behaviour), except during TV watching. More than 10% of images during TV watching contained eating and drinking activities (meal, snack, and beverage). Details of co-existing behaviour can be seen in Appendix D, Table D4.

### 3.8 Multiscreens

The use of multiple screens was seen in 2526 images (12.7% of all images), with the majority being a combination of smartphone and TV (99.4%), and the rest was a combination of smartphone and laptop. Details of the use of multiscreens can be seen in Appendix D, Table D5. From interview data, it appeared that participants used multiscreens either as a secondary device, such as using their smartphone when there were advertisements on TV or as a background.

“The TV was on (as a background) so that it won't be quiet (laughing)” **(PF3)**.

“I watched TV while checking my phone” **(PF4).**

### 3.9 Themes based on interviews

We generated four themes related to participants’ physical activity and sedentary behaviour based on interviews:

1. Participants' awareness to follow the current physical activity and sedentary behaviour guidelines for adolescents seemed not visible.

The majority of participants did not allocate specific time for structured physical activity on either school days and non-school days and did not meet the current WHO physical activity guidelines for adolescents. Participants reported doing some structured physical activity during the weekend, but only when not feeling busy. They did some physical activity in the form of domestic work for helping their parents.

*“(On weekend days) if I have free time and if my Mom didn't ask me to go with her to the market, I usually ride a bicycle”***(PF5).**

*“In the morning, usually I helped my Mom. Mm … tidying up my bed, then sweeping the floor, tidying up a bookshelf, then taking care of my little brother”***(PF1).**

In addition, all participants admitted that they did high amounts of screen time every day and yet seemed to show minimal concern. These situations reflected that participants were either unaware of the need to follow the current physical activity and sedentary behaviour guidelines for adolescents, or were unaware of such guidelines, or the importance to follow the guidelines for their overall health and wellbeing.

2. Screen-based sedentary behaviour appeared to be an automatic behaviour during participants' free time.

Screen time seemed to be an integral part of participants' lives in that they allocated much time for this behaviour whenever possible across the day. This was for various reasons, such as educational, social, and leisure reasons. The feeling of urgency to check social media seemed to increase participants’ screen time significantly.

*“Because there were a lot of assignments. So I have to check them (on WhatsApp), what is the information”***(PF2).**

*“There were incoming information from (WhatsApp) group, so I checked my phone”***(PF1)**

While participants were aware of doing ‘excessive’ screen time, it seemed hard for them to reduce it because they were too excited with that activity or had no other activity options. They just do screen time ‘automatically’ during leisure time.

*“I think I used it (smartphone) for too long, but if I have to reduce it, it is the only entertainment I have. What else do I have to do?“***(PF3)**.

3. The lack of parents' supervision seemed to facilitate longer recreational screen time

Interviews revealed that minimum supervision from parents appeared to cause participants to do longer recreational screen time. All participants cited that their parents or other family members rarely reminded them to reduce screen time and that the control to stop screen time was mainly from participants’ self-awareness. Participants also reported that there is no specific limitation on the permitted duration for recreational screen time and this appeared to facilitate longer screen time.

*“(I stop using screen-based devices) because I feel bored and tired. My parents rarely reminded me”***(PF1)**.

*“(I stop using smartphone) after finishing looking for information. (I: Did your parents ask you to stop using phone?) No, I stop it by myself’”***(PF5*)****.*

4. Dual positive and negative effects of screen time

Participants are already aware of both the positive and negative effects of screen time. They acknowledged the benefits of screen time for studying, recreation, communication, and getting information.

*“The benefits (of screen time) are for refreshing, for recreation, so I won't be too dizzy. I got some information as well. When I scrolled my Instagram I got the information, for communication too …“***(PF3).**

But they also felt some drawbacks from screen time, including mental and/or physical health problems, disrupted sleep, study-related problems, and bad time management. Regarding mental health, some participants reported experiencing some emotional disturbance due to getting bad comments on social media. Other participants cited physical health problems due to excessive screen time, such as headaches, sore eyes, and feeling tired.

*“Yes, (screen time) disturbed my bedtime, sometimes I felt sore eyes”***(PF1).**

*“Because I used it (smartphone) for too long, it was like … I forgot to eat, I mean I am late to eat, late to pray. Then I felt lazy to study. … when I used it too long, I felt dizzy, unwell, so I stop it”***(PF2).**

## 4 Discussion

Our study aimed to investigate physical activity and sedentary behaviour by using a detailed and comprehensive multi-method approach in a small group of Indonesian female adolescents. We aimed to achieve an enhanced understanding of these behaviours, contexts, and likely influences and perceptions, but not to offer generalizability. That requires a larger sample. Here, we will discuss our method and findings related to duration, patterns, and contexts of physical activity and sedentary behaviour.

The use of a multi-method approach in our study has facilitated a rich and comprehensive understanding of the duration, patterns and contexts of physical activity and sedentary behaviour. The approach also increased our understanding of the reasons why participants performed certain behaviours. Moreover, the approach enabled us to triangulate findings from different types of measurement. The most apparent drawback of this approach is that it is somewhat intrusive and time-consuming. However, this approach is also considered highly useful and worthwhile to understand behaviour in a more comprehensive way. Future studies might consider using similar methods in a larger sample.

Consistent with Guthold et al.‘s study,^17^ in our small sample, we found 4 out of 5 participants did not meet the WHO's physical activity guidelines for adolescents.^38^ Based on interviews, these results seemed partly related to the lack of knowledge of the guidelines and the lack of awareness of the importance to follow the guidelines. The low level of moderate to vigorous physical activity among participants may also be associated with the perceived importance of life priorities in this age, which is primarily for education and study.^39^ Furthermore, adolescence is a pubertal stage, a period for a growth spurt that includes significant alterations to physical, sexual, cognitive, social, and emotional development due to hormonal changes.^40^^,^^41^ These changes may partly affect adolescents' physical activity. Metcalf et al.^42^ found that the pubertal stage caused a more rapid reduction in physical activity in female adolescents. A combination of physical and psychosocial change is likely to be a cause, as well as the influence of sociocultural norms in Indonesia. This highlights the importance of studies emanating from a diverse range of countries, including LMICs.

In line with previous studies^17^^,^^43^^,^^44^ but especially on non-school days, our research found that participants’ physical activity was dominated by light intensity. It may be beneficial for future physical activity interventions to include light physical activity. Del Pozo Cruz et al.^45^ pointed out that due to the low prevalence of moderate-to-vigorous physical activity in the population, promotion of physical activity may take advantage of rising evidence of the benefit of light physical activity, such as for reducing depressive symptoms.^46^

Similar to previous studies,^47^^,^^48^ we found that participants spent a great proportion of their time on screen-based sedentary behaviour. Our interviews revealed that the high amount of screen use, in part, seemed to be influenced by the feeling of urgency to check information, including schoolwork. In this digital era, it is not uncommon for adolescents to work on school assignments and discuss them with friends using screen-based devices. This contributes to the need for screen use. A recent study showed that educational demands increased adolescents' screen time significantly.^49^ Moreover, the availability of screen-based devices, as well as internet connection, supported adolescents to engage in more screen time.^19^^,^^49^ Various functions that can be served by screen-based devices, such as for social (e.g., communicate with friends through WhatsApp), entertainment (e.g., watching videos on YouTube), and functional purposes (e.g., browsing the internet), also seem to trigger screen use.^19^ Our interview data showed that the lack of parental supervision seemed to facilitate longer recreational screen time. A recent study also found that control from parents appeared to influence the duration of screen time in adolescents.^49^ Future work will need to ascertain the risks and benefits of recreational screen time when seen alongside ‘productive’ school-based screen use.

Similar to a recent wearable camera study,^48^ we found that participants allocated around one-third of smartphone use for social media activity. This finding appeared to be associated with one of the primary psychosocial tasks that must be achieved by adolescents, that is to secure social connections and to obtain recognition from peers,^50^ which trigger them to stay updated with peers through social media apps. Some have argued that concerns about psychosocial outcomes of excessive use of social media are not straight forward, but certainly more work is needed on these more recent platforms.^51^ We claim to make a small contribution in addressing Orben's concern that the literature is a “mostly stagnating and conflicting research area”^51^ by using a diverse multi-method approach.

Our study revealed that participants’ screen time was dominated by passive use of screens. This finding is in line with previous literature.^48^^,^^52^ While the different effects between interactive and passive screen time still need to be studied further, some evidence has shown that passive screen time seems to be associated with more detrimental effects than interactive uses.^32^^,^^52^^,^^53^ Interactive screen time can involve some physical exercise that may bring benefits toward physical and mental health, and may also include cognitive engagement and, hence, possible cognitive development and improved cognitive functioning.^32^ A recent study found that interactive screen time correlates with positive educational.^52^ In contrast, evidence showed that passive screen time correlates with worse psychological outcomes, such as mood and anxiety disorders,^52^^,^^53^ poorer health outcomes, and lower educational outcomes.^52^

Consistent with findings from previous studies,^48^^,^^54^ we found that participants did screen-based sedentary behaviour mainly in the evening on school days and in the morning on weekend days/holidays. Having more opportunities to access screen-based devices during those time frames seemed to be one of the main reasons. Many cartoon programs that were favoured by participants were aired on weekend mornings. The target for modifying screen-based sedentary behaviour in female adolescents may focus on those time frames. Limiting access to screen-based devices and providing alternative activities other than screen time could become options. This will require better understanding of the temporal patterning of such behaviours (hence our assessment using cameras), and what behaviours realistically compete for the attention of the adolescents at different time periods across the day. We argued some years ago, that TV may not be so problematic later in the evening when physically active behaviours are unlikely or even not possible.^55^

Additionally, our study found that screen-based sedentary behaviour in the form of a smartphone was mostly done alone in the bedroom or living room. The desire to have the freedom to access content appeared to be the reason for these findings. A previous study found a similar result that adolescents used smartphones ultimately in the bedroom as they did not want to be interrupted and wanted some privacy for their screen use.^48^ Among characteristics that emerge during the adolescent stage are the need to have more privacy and to pursue more autonomy from family.^41^^,^^50^ This may explain our result of why participants spent more time in solitary contexts when using screen-based devices.

Our camera data showed that more than a quarter of movement behaviour consist of incidental physical activity, such as domestic chores. Promoting incidental physical activity for female adolescents can be beneficial to increase their physical activity.^56^ Unlike structured exercise, incidental physical activity has advantages, including not requiring specific time allocation and can be performed as part of daily activities, such as walking from place to place, climbing stairs, and doing active domestic tasks.^56^

Our wearable camera data showed that non-screen-based sedentary behaviour comprised just over 10% of total images, and this behaviour was mainly done for educational purposes, such as studying and working on assignments. To date, we did not find any recent wearable camera study that investigate non-screen-based sedentary behaviour. A previous study investigating non-screen based sedentary behaviour was done in 2010.^57^ That study used activity recall and found that adolescents spent 60% of their sedentary time on non-screen-based activities.^57^ However, it seems irrelevant to compare the result of that study with ours because of the different method and timing. In 2010, the use and the availability of screen-based devices as well as internet connection was much different than now.

The vast majority of sedentary behaviour studies have mainly focus on screen-based sedentary behaviour. Future studies need to investigate the different effects between screen- and non-screen-based sedentary behaviour toward various health and developmental outcomes.

### 4.1 Strengths and limitations

The key strength of our study includes the multi-method approach that was used, involving both self-report and modern technology-based measurements as well as quantitative and qualitative methods (accelerometers, automated wearable cameras, diaries, and interviews). Unlike previous studies, ours covers a broad spectrum of physical activity as well as screen- and non-screen-based sedentary behaviour which enable us to understand female adolescent behaviours more comprehensively. We triangulated the results from device-based measurements with diary and interview data to further elaborate on our findings. Our study also adds to the limited body of evidence concerning these issues in LMICs.

A limitation of our study is the small sample size. However, our study did not aim to generalize our findings to Indonesians or other populations. We sought multi-methodological diversity. Another limitation is that we collected data on non-PE school days, thus may have underestimated physical activity levels. Participants also wore the accelerometers significantly longer during school time than after school. These should be noted when interpreting the results. Moreover, participants may change their behaviour during data collection, such as spending more time at home because of wearing the automated wearable camera. The camera was only able to capture events in front of participants so that we may miss identifying other people or family members in close proximity. We were not able to identify the content of screen-based devices being used when they were out of frame. Participants may fail to report some activities when they removed the devices during data collection. These situations may have affected our study results.

## 5 Conclusions

From our small sample, the research found that participants spent a great proportion of their time on screen-based sedentary behaviour, which was dominated by passive screen use. Screen time was mainly done on school day evenings and on non-school day mornings. Smartphone use was mostly done in the bedroom and living room and in a solitary context. Interviews suggest that the high amount of screen time seemed to be influenced by a lack of awareness of current guidelines, the feeling of urgency to check information, and the lack of parental supervision. The use of the multi-method approach has facilitated a better and more comprehensive understanding of the duration, patterns, and contexts of physical activity and sedentary behaviour in participants.

## Author contributions

F.D.A.: Conceptualization, Methodology, Formal analysis, Resources, Project administration, Ethics Application, Visualisation, Investigation, Writing—original draft Writing—review and editing. S.J.H.B: Conceptualization, Methodology, Ethics Application, Writing—review and editing, Supervision. A.A.P: Formal analysis, Investigation, Writing—review and editing. G.T.: Resources, Ethics Application, Writing—review and editing. K.D.C.: Conceptualization, Methodology, Ethics Application, Writing—review and editing, Supervision.

## Funding

F.D.A. is supported by the Australia Awards Scholarship (Grant ID: ST000TB39).

## Declaration of competing interest

The authors have no conflicts of interest relevant to this article.

## Supplementary data

Appendix A Coding Guideline for Automated Wearable Camera Data Appendix A

Appendix B Interview Guideline Appendix B

Appendix C Table C1: Patterns of activity during school and non-school days using accelerometer data; Table C2: Patterns of activity during school and non-school days using camera data. Appendix C

Appendix D Table D1: Purpose of activity based on camera data; Table D2: Physical setting of activity based on camera data; Table D3: Social context, environment, and interaction based on camera data; Table D4: Co-existing behaviour based on camera data; Table D5: Multiscreens based on camera data Appendix D
