# Supplementary material for: Physical activity and sedentary behaviour of female adolescents in Indonesia: A multi-method study on duration, pattern and context
Source: J Exerc Sci Fit. 2022 Feb 24;20(2):128–39. doi: 10.1016/j.jesf.2022.02.002 (PMC8899402; doi:10.1016/j.jesf.2022.02.002)
Supplement: Appendix C — Table C1: Patterns of activity during school and non-school days using accelerometer data; Table C2: Patterns of activity during school and non-school days using camera data. [file mmc3.docx]

**APPENDIX C**

**Table C1.** **Patterns of activity during school and non-school days using accelerometer data**

School days

| **% of wear time** | **before 700h** | **700h-1500h** | **1500h-1800h** | **1800h-sleep** |
| --- | --- | --- | --- | --- |
|  | M (SD) | M (SD) | M (SD) | M (SD) |
| SB | 5.4 (1.8) | 41.1 (4.5) | 10.5 (7.5) | 13.1 (2.6) |
| LPA | 2.7 (1.0) | 16.2 (2.1) | 3.0 (2.1) | 2.4 (1.5) |
| MPA | 0.1 (0.1) | 0.6 (0.3) | 0.2 (0.2) | 0.3 (0.4) |
| VPA | 0.0 (0.0) | 0.0 (0.0) | 4.6 (10.3) | 0.0 (0.0) |
|  |  |  |  |  |

Non-school days

| **% of wear time** | **before 1200h** | **1200h-1500h** | **1500h-1800h** | **1800h-sleep** |
| --- | --- | --- | --- | --- |
|  | M (SD) | M (SD) | M (SD) | M (SD) |
| SB | 34.9 (15.3) | 22.8 (24.1) | 10.8 (7.7) | 13.1 (10.4) |
| LPA | 6.8 (1.3) | 3.2 (3.3) | 1.5 (1.4) | 5.7 (7.8) |
| MPA | 0.3 (0.6) | 0.2 (0.3) | 0.1(0.1) | 0.4 (0.7) |
| VPA | 0.0 (0.0) | 0.0 (0.0) | 0.0 (0.0) | 0.0 (0.0) |

**Table C2.** **Patterns of activity during school and non-school days using camera data**

School days

| **% Number of Images** | **15.00h - 18.00h** | **18.00h - sleep** |
| --- | --- | --- |
|  | **M (SD)** | **M (SD)** |
| PA images | 0.4 (0.4) | 4.4 (8.3) |
| Screen-based PA images | 0.0 (0.1) | 1.3 (2.9) |
| Screen-based SB images | 13.6 (23.1) | 44.4 (33.7) |
| Non-screen-based SB images | 0.7 (0.8) | 31.6 (34.8) |

Non-school days

| **% Number of Images** | **before 12.00h** | **12.00h - 15.00h** | **15.00h - 18.00h** | **18.00h - sleep** |
| --- | --- | --- | --- | --- |
|  | **M (SD)** | **M (SD)** | **M (SD)** | **M (SD)** |
| PA images | 2.8 (3.0) | 0.8 (1.1) | 0.2 (0.2) | 4.0 (7.7) |
| Screen-based PA images | 0.2 (0.3) | 1.1 (2.2) | 0.3 (0.5) | 0.1 (0.2) |
| Screen-based SB images | 23.7 (22.0) | 21.0 (21.9) | 7.5 (6.1) | 15.4 (14.7) |
| Non screen-based SB images | 4.9 (5.3) | 8.3 (16.1) | 1.4 (1.5) | 0.9 (1.4) |
