# Supplementary material for: Physical activity and sedentary behaviour of female adolescents in Indonesia: A multi-method study on duration, pattern and context
Source: J Exerc Sci Fit. 2022 Feb 24;20(2):128–39. doi: 10.1016/j.jesf.2022.02.002 (PMC8899402; doi:10.1016/j.jesf.2022.02.002)
Supplement: Appendix D — Table D1: Purpose of activity based on camera data; Table D2: Physical setting of activity based on camera data; Table D3: Social context, environment, and interaction based on camera data; Table D4: Co-existing behaviour based on camera data; Table D5: Multiscreens based on camera data [file mmc4.docx]

**Appendix D**

**Table D1. Purpose of activity based on camera data**

| **Purpose** | ***n* of images** | **%** |
| --- | --- | --- |
| **Physical Activity** |  |  |
| Leisure | 120 | 11.8 |
| Other | 120 | 11.8 |
| Unclassifiable | 469 | 46.2 |
| Personal Care | 24 | 2.4 |
| Domestic work | 283 | 27.9 |
| **SUM** | **1016** | **100.0** |
| **Non-screen based SB** |  |  |
| Educational | 954 | 44.3 |
| Leisure | 387 | 18.0 |
| Other | 203 | 9.4 |
| Unclassifiable | 299 | 13.9 |
| Personal Care | 162 | 7.5 |
| Social | 150 | 7.0 |
| **SUM** | **2155** | **100.0** |
| **Screen-based SB** |  |  |
| **Portable >Mobile Device (phone,ipad)** | |  |
| Leisure | 3808 | 30.1 |
| Other | 0 | 0.0 |
| Unclassifiable | 6361 | 50.3 |
| Social | 2060 | 16.3 |
| Educational | 421 | 3.3 |
| **SUM** | **12650** | **100.0** |
| **Portable >Laptop Computer** | |  |
| Unclassifiable | 85 | 22.5 |
| Educational | 292 | 77.5 |
| **SUM** | **377** | **100.0** |
| **Non-portable >Television** | |  |
| Leisure | 2563 | 98.5 |
| Unclassifiable | 38 | 1.5 |
| **SUM** | **2601** | **100.0** |

**Table D2. Physical setting of activity based on camera data**

| **Physical Setting** | ***n* of images** | **%** |
| --- | --- | --- |
| **Physical Activity** |  |  |
| Home >Bedroom | 403 | 39.7 |
| Home >Living room | 251 | 24.7 |
| Home >Outside | 4 | 0.4 |
| Home >Other (e.g., Office, bathroom) | 54 | 5.3 |
| Home >Kitchen/Dining Room | 200 | 19.7 |
| Home >Unclassifiable | 85 | 8.4 |
| Public >Street | 7 | 0.7 |
| School >Other (e.g., Office) | 12 | 1.2 |
| **SUM** | **1016** | **100.0** |
| **Non-screen based SB** |  |  |
| Home >Bedroom | 1427 | 66.2 |
| Home >Living room | 593 | 27.5 |
| Home >Outside | 111 | 5.2 |
| Home >Other (e.g., Office, bathroom) | 2 | 0.1 |
| Home >Kitchen/Dining Room | 6 | 0.3 |
| Home >Unclassifiable | 16 | 0.7 |
| **SUM** | **2155** | **100.0** |
| **Screen-based SB** |  |  |
| **Portable >Mobile Device (smartphone)** | |  |
| Home >Bedroom | 6391 | 50.5 |
| Home >Living room | 5675 | 44.9 |
| Home >Outside | 0 | 0.0 |
| Home >Other (e.g., Office, bathroom) | 0 | 0.0 |
| Home >Kitchen/Dining Room | 0 | 0.0 |
| Home >Unclassifiable | 12 | 0.1 |
| Public >Street | 0 | 0.0 |
| School >Other (e.g., Office) | 572 | 4.5 |
| **SUM** | **12650** | **100.0** |
| **Portable >Laptop Computer** | |  |
| Home >Bedroom | 260 | 69.0 |
| School >Other (e.g., Office) | 117 | 31.0 |
| **SUM** | **377** | **100.0** |
| **Non-portable >Television** | |  |
| Home >Bedroom | 1036 | 39.8 |
| Home >Living room | 1565 | 60.2 |
| **SUM** | **2601** | **100.0** |

**Table D3. Social context, environment, and interaction based on camera data**

| **Variable** | ***n* of images** | **%** |
| --- | --- | --- |
| **Physical Activity** |  |  |
| **Social Context** |  |  |
| Alone | 903 | 88.9 |
| Direct social engagement | 12 | 1.2 |
| Social environment but no interaction | 101 | 9.9 |
| **Social Environment** |  |  |
| Alone (i.e., none) | 903 | 88.9 |
| Adult & child | 78 | 7.7 |
| Child | 21 | 2.1 |
| Adult | 7 | 0.7 |
| Unclassifiable | 7 | 0.7 |
| **Social Interaction** |  |  |
| None | 912 | 89.8 |
| Conversation | 6 | 0.6 |
| Background | 24 | 2.4 |
| Co-viewing | 71 | 7.0 |
| Unclassifiable | 3 | 0.3 |
| **Non-screen-based SB** |  |  |
| **Social Context** |  |  |
| Alone | 1774 | 82.3 |
| Direct social engagement | 164 | 7.6 |
| Social environment but no interaction | 217 | 10.1 |
| **Social Environment** |  |  |
| Alone (i.e., none) | 1774 | 82.3 |
| Adult & child | 187 | 8.7 |
| Child | 152 | 7.1 |
| Adult | 38 | 1.8 |
| Unclassifiable | 4 | 0.2 |
| **Social Interaction** |  |  |
| None | 1774 | 82.3 |
| Conversation | 160 | 7.4 |
| Background | 217 | 10.1 |
| Other | 4 | 0.2 |
| **Screen-based SB** |  |  |
| **Portable >Mobile Device (smartphone)** |  |  |
| **Social Context** |  |  |
| Alone | 9234 | 73.0 |
| Direct social engagement | 47 | 0.4 |
| Social environment but no interaction | 3369 | 26.6 |
| **Social Environment** |  |  |
| Alone (i.e., none) | 9234 | 73.0 |
| Adult & child | 1040 | 8.2 |
| Child | 1034 | 8.2 |
| Adult | 1039 | 8.2 |
| Unclassifiable | 303 | 2.4 |
| **Social Interaction** |  |  |
| None | 9677 | 76.5 |
| Conversation | 47 | 0.4 |
| Background | 1980 | 15.7 |
| Co-viewing | 938 | 7.4 |
| Unclassifiable | 8 | 0.1 |
| **Portable >Laptop Computer** |  |  |
| **Social Context** |  |  |
| Alone | 377 | 100 |
| **Social Environment** |  |  |
| Alone (i.e., none) | 377 | 100 |
| **Social Interaction** |  |  |
| None | 377 | 100 |
| **Non-portable >Television** |  |  |
| **Social Context** |  |  |
| Alone | 1603 | 61.6 |
| Direct social engagement | 33 | 1.3 |
| Social environment but no interaction | 965 | 37.1 |
| **Social Environment** |  |  |
| Alone (i.e., none) | 1603 | 61.6 |
| Adult & child | 309 | 11.9 |
| Child | 242 | 9.3 |
| Adult | 214 | 8.2 |
| Unclassifiable | 233 | 9.0 |
| **Social Interaction** |  |  |
| None | 1603 | 61.6 |
| Conversation | 1 | 0.0 |
| Background | 28 | 1.1 |
| Co-viewing | 959 | 36.9 |
| Unclassifiable | 10 | 0.4 |

**Table D4. Co-existing behaviour based on camera data**

| **Co-existing Behaviour** | ***n* of images** | **%** |
| --- | --- | --- |
| **Physical Activity** |  |  |
| None | 1000 | 98.4 |
| Food >Meal | 8 | 0.8 |
| Food >Snack | 8 | 0.8 |
| **Non-screen based SB** |  |  |
| None | 2135 | 99.1 |
| Food >Meal | 5 | 0.2 |
| Food >Snack | 12 | 0.6 |
| Food >Other | 2 | 0.1 |
| Food >Unclassifiable | 1 | 0.0 |
| **Screen-based SB** |  |  |
| **Portable >Mobile Device (phone,ipad)** | |  |
| None | 12621 | 99.8 |
| Multitask >Book or paper (e.g., Reading) | 2 | 0.0 |
| Food >Snack | 3 | 0.0 |
| Multitask >Other | 1 | 0.0 |
| Multitask >Pen and Paper (e.g., Writing) | 1 | 0.0 |
| Food >Other | 15 | 0.1 |
| Multitask >Book (e.g., Reading) | 7 | 0.1 |
| **Portable >Laptop Computer** | |  |
| None | 377 | 100% |
| **Non-portable >Television** | |  |
| None | 2282 | 87.7 |
| Multitask >Book or paper (e.g., Reading) | 2 | 0.1 |
| Food >Snack | 45 | 1.7 |
| Food >Meal | 239 | 9.2 |
| Drink >Beverage | 33 | 1.3 |

**Table D5. Multiscreen based on camera data**

| **Multiscreen** | ***n* of images** | **%** |
| --- | --- | --- |
| Non-portable >Television + smartphone | 2512 | 99.4 |
| Portable >Laptop Computer+smartphone | 14 | 0.6 |
| SUM | 2526 | 100.0 |
